# Deregulation of ATG9A by impaired AR signaling induces autophagy in prostate stromal fibroblasts and promotes BPH progression

**DOI:** 10.1038/s41419-018-0415-2

**Published:** 2018-03-22

**Authors:** Chen-Yi Jiang, Bo-Yu Yang, Sheng Zhao, Si-Hui Shao, Xiao-Yu Bei, Fei Shi, Qian Sun, Zheng Deng, Xiao-Hai Wang, Bang-Min Han, Fu-Jun Zhao, Shu-Jie Xia, Yuan Ruan

**Affiliations:** 1 Department of Urology, Shanghai General Hospital, Shanghai Jiao Tong University School of Medicine, Shanghai, 200080 China; 2 Hangzhou Normal University School of Medicine, Hangzhou, 311121 China; 3 Department of Urology, Shanghai General Hospital of Nanjing Medical University, Shanghai, 200080 China

## Abstract

The activation of androgen receptor (AR) signaling plays an essential role in both prostate stromal cells and epithelial cells during the development of benign prostatic hyperplasia (BPH). Here we demonstrated that androgen ablation after 5α-reductase inhibitor (5-ARI) treatment induced autophagy in prostate stromal fibroblasts inhibiting cell apoptosis. In addition, we found that ATG9A expression was increased after androgen ablation, which facilitated autophagic flux development. Knockdown of ATG9A not only inhibited autophagy notably in prostate stromal fibroblasts, but also reduced the volumes of prostate stromal fibroblast and epithelial cell recombinant grafts in *nude* mice. In conclusion, our findings suggested that ATG9A upregulation after long-term 5-ARI treatment constitutes a possible mechanism of BPH progression. Thus, combined treatment with 5-ARI and autophagy inhibitory agents would reduce the risk of BPH progression.

## Introduction

Benign prostatic hyperplasia (BPH), a highly prevalent and progressive disease, is commonly associated with lower urinary tract symptoms (LUTS) and greatly affects the patients’ quality of life^1^. The prostate is a hormone-responsive organ, and androgens play essential roles in its development, as well as in the maintenance of tissue function and even the pathogenesis of prostatic diseases^2^. Androgens mediate proliferation and differentiation in both epithelial cells and prostate stromal fibroblasts^3^. Dihydrotestosterone (DHT), the primary prostatic androgen, is transformed from testosterone by type II 5α-reductase in prostate cells^4^. Inhibition of 5α-reductase function with 5α-reductase inhibitors (5-ARIs), such as finasteride and dutasteride, impairs androgen receptor (AR) signaling via ablating DHT formation and induces apoptosis in prostate epithelial cells, which is the basis for BPH therapy^5^. It is known that finasteride treatment reduces intra-prostatic DHT levels in BPH patients by approximately 85%^6^. Clinical trials have confirmed that 5-ARI alone or combined with α-1 blockers could effectively decrease LUTS and the risk of BPH progression^7^. Our previous study, however, found that more than 24% of patients on 5-ARIs undergo transurethral resection of the prostate due to clinical progression of BPH^8^. Other reports also pointed out that there are still more than 17% of patients suffering from BPH progression after long-term 5-ARI treatment^9,10^. Meanwhile, the effects of 5-ARIs on prostate stromal fibroblasts remain unclear. Exploring the molecular and cellular mechanisms involved in the response of prostate stromal fibroblasts after long-term application of 5-ARIs would help develop more efficient approaches for the prevention of BPH progression.

Autophagy is a highly conserved evolutionary and complex cellular process in eukaryotic cells, where cytoplasmic long half-life proteins and organelles are sequestered within autophagosomes and delivered to lysosomes for degradation and recycling^11^. Previous studies have revealed that autophagy is involved in BPH development^12,13^. In addition, numerous studies provided evidence that AR signaling participates in the process of autophagy. Li et al. reported that androgen inhibition induces autophagy in prostate epithelial cells^14^; meanwhile, castration therapy in prostatic cancer also induces autophagy in cancer cells, which could escape death^15,16^. Based on these findings, we hypothesized that impairment of AR signaling by long-term 5-ARI treatment may contribute to autophagy induction, which increases the viability of prostate stromal fibroblasts and promotes BPH progression.

In this study, we measured the autophagy levels of BPH tissues, and found that impaired AR signaling following 5-ARI treatment could induce autophagy in prostate stromal fibroblasts. Furthermore, autophagy PCR array was performed to screen differentially expressed autophagy-related (ATG) genes in prostate stromal fibroblasts after treatment with different DHT concentrations. Finally, we found that ATG9A, which plays a pivotal role in regulating autophagy initiation, was upregulated after androgen ablation in prostate stromal fibroblasts. These findings suggest that autophagy induced by deregulated ATG9A in prostate stromal fibroblasts after androgen ablation could be a possible reason for BPH progression.

## Results

### Autophagy is upregulated in BPH stromal fibroblasts

**Fig. 1:**
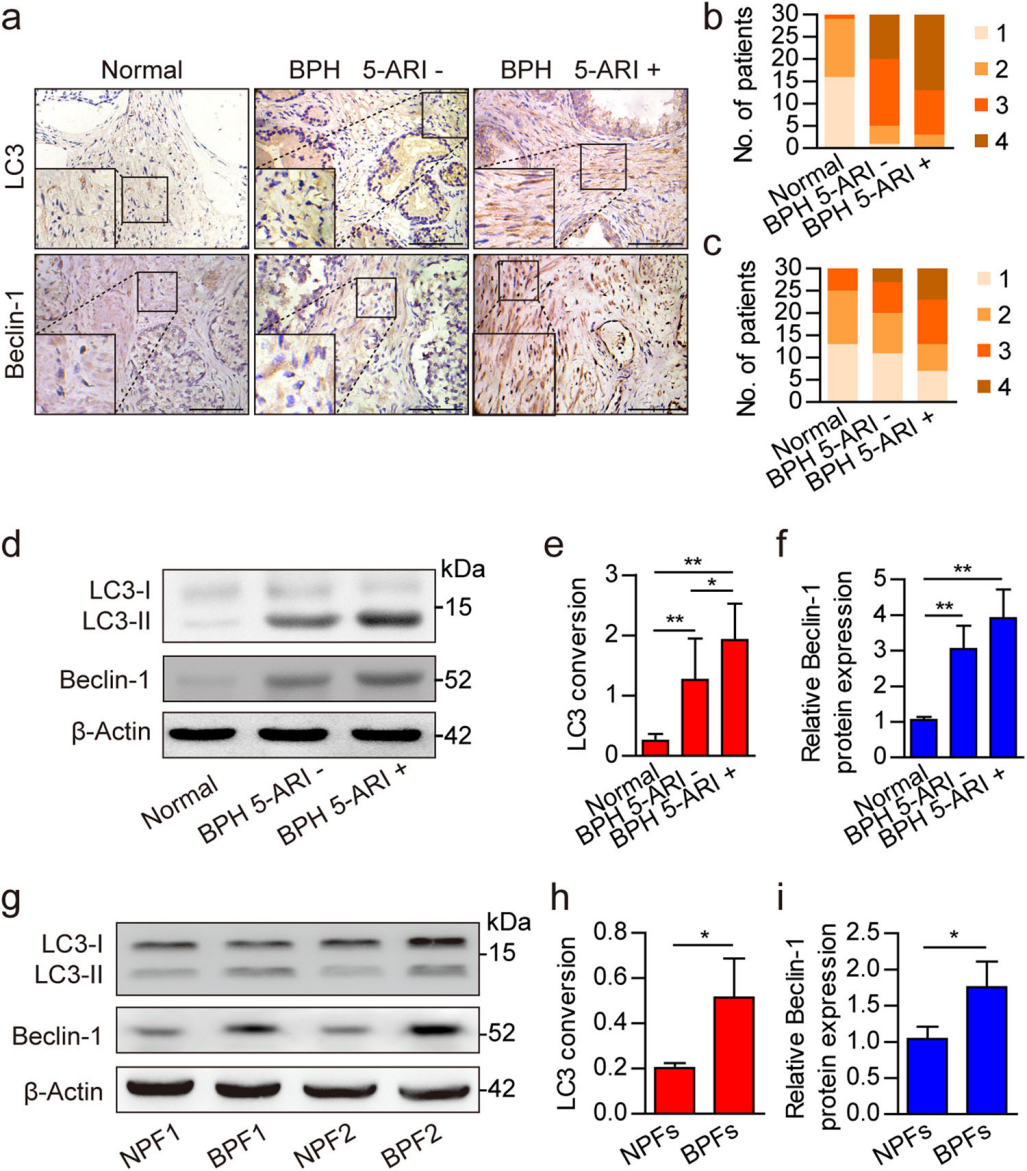
Expression levels of autophagy-related proteins in normal prostate and BPH tissues. **a** Immunohistochemistry results showing LC3 and Beclin-1 expression levels in normal prostate and BPH tissues after 5-ARI treatment (5-ARI+) or without 5-ARI treatment (5-ARI−). Scale bar, 100 μm. **b**, **c** Bar graphs showing patient stratification by stromal LC3 and Beclin-1 expression scores in each group. **d** Western blot data showing the LC3 conversion ratio (LC3-II/β-Actin) (**e**) and relative Beclin-1 expression levels (**f**) in normal prostate and BPH tissues with or without 5-ARI treatment. **g** Western blot data showing the LC3 conversion ratio (LC3-II/β-Actin) (**h**) and relative Beclin-1 expression levels (**i**) in normal prostate fibroblasts (NPFs) and BPH derived prostate fibroblasts (BPFs). *P < 0.05, **P < 0.01

Our previous work found a relation between autophagy level in the prostatic stroma and BPH severity^17^. However, the effects of autophagy on BPH stromal fibroblasts remain unknown and need further investigation. To determine the autophagy level in prostate stromal fibroblasts, human prostate samples were divided into three groups: normal prostate, BPH without 5-ARI treatment, and BPH with 5-ARI treatment. Then, expression levels of the autophagy-related proteins LC3 and Beclin-1 were analyzed by immunohistochemistry (IHC) (Fig. 1a). The results showed that stromal expression levels of LC3 and Beclin-1 were higher in BPH tissue samples compared with normal prostate specimens, indicating a possible increase of autophagy level in the BPH stroma (Fig. 1b, c, and Supplementary Table 1). Furthermore, compared with other groups, protein extracts from BPH with 5-ARI treatment showed higher LC3 conversion ratio (Fig. 1d, e). Besides, Beclin-1 expression levels were higher in BPH groups compared with the normal prostate (Fig. 1d, f). To examine the basal autophagy level in primary prostate stromal fibroblasts, that were isolated from BPH (BPFs) and normal prostate (NPFs). As shown in Fig. 1g–i, both LC3 conversion ratio and Beclin-1 expression levels were higher in BPFs compared with NPFs. These results indicated that the suppression of AR signaling by 5-ARI treatment induced autophagy in the prostatic stromal fibroblasts.

### Impaired AR signaling induces autophagy in prostate stromal fibroblasts

**Fig. 2:**
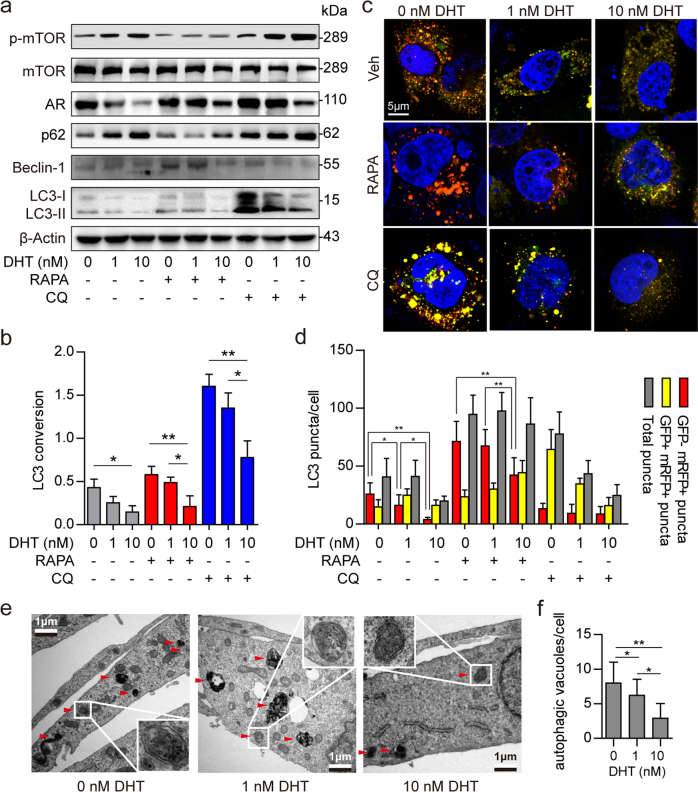
Androgen ablation induces autophagy in prostate stromal fibroblasts. **a** Western blot data showing phospho-mTOR (p-mTOR), mTOR, AR, p62, and Beclin-1 expression levels and the LC3 conversion ratio (LC3-II/β-Actin) **b** in WPMY-AR cells. The cells were cultured with 0, 1, and 10 nM DHT, respectively, for 48 h, and treated with 50 nM RAPA or 50 μM CQ for 3 h before protein extraction. **c**, **d** WPMY-AR cells were infected with mRFP–GFP–LC3 adenovirus and cultured with 0, 1, and 10 nM DHT, respectively, for 48 h, and treated with 50 nM RAPA or 50 μM CQ for 3 h before 4% paraformaldehyde fixation and DAPI counterstaining. Scale bar, 5 μm. Bar graph showing LC3 puncta formation in different groups. **e**, **f** Transmission electron micrographs for detecting autophagic vacuoles in WPMY-AR cells cultured with 0, 1 and 10 nM DHT for 48 h, respectively, and treated with 50 nM RAPA for 3 h before 2% glutaraldehyde fixation. Enlarged images show double-membrane autophagic vacuoles. Scale bar, 1 μm. *P < 0.05, **P < 0.01

To test our hypothesis that impaired AR signaling induces autophagy in prostate stromal fibroblasts, we engineered prostate stromal fibroblasts that stably overexpress AR (WPMY-AR), and assessed autophagy level alteration in these cells under different concentrations of DHT. As shown in Fig. 2a, b, basal autophagy was suppressed with WPMY-AR cultured in medium containing DHT, in a concentration-dependent manner. After cells exposure to the mTOR inhibitor rapamycin (RAPA) for 3 h, the LC3 conversion ratio and Beclin-1 protein levels in WPMY-AR were remarkably increased under 0 nM DHT and slightly elevated under 1 nM DHT, compared with the 10 nM DHT group. Meanwhile, p62 protein levels were increased in a DHT concentration-dependent manner. These findings indicated that DHT can inhibit not only basal autophagy but also RAPA-induced autophagy in prostatic fibroblasts. More importantly, 10 nM DHT still inhibited autophagy when RAPA was applied to inhibit mTOR activation. This suggested that in addition to affecting mTOR activation, AR suppression also induced autophagy in an mTOR-independent manner. Next, the mRFP–GFP–LC3 adenovirus system further confirmed the high autophagy levels in cells cultured in presence of low concentrations of DHT. As shown in Fig. 2c, d, both yellow and red LC3 puncta were accumulated with decreasing DHT concentrations. Treating the cells with 50 nM RAPA further induced red LC3 puncta accumulation, while 50 μM chloroquine (CQ) treatment inhibited red LC3 puncta conversion. In addition, the formation of autophagosome and autolysosome was monitored under different DHT concentrations plus 50 nM RAPA in WPMY-AR by transmission electron microscopy (TEM). As shown in Fig. 2e, f, more autophagic vacuoles in WPMY-AR cells were found at 0 and 1 nM DHT compared with the 10 nM DHT group. These findings suggested that impaired AR signaling by androgen ablation induced autophagy in prostate stromal fibroblasts.

### Autophagy induction attenuates apoptosis in prostate stromal fibroblasts

**Fig. 3:**
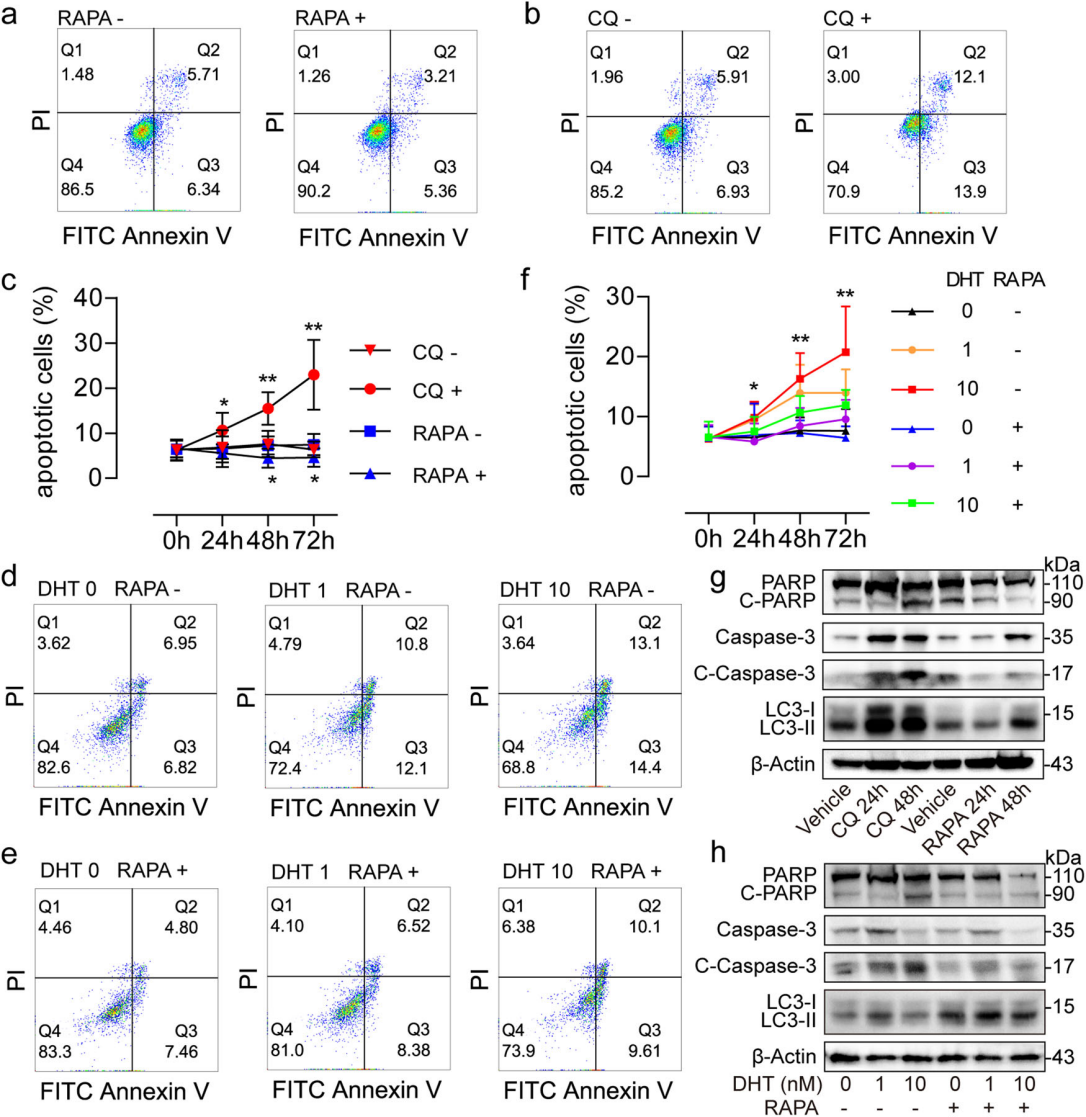
Apoptosis analyses in prostate stromal fibroblasts. **a** Annexin V/PI flow cytometry results showing different apoptotic rates of WPMY-1 cells treated with 50 nM RAPA (RAPA+) or without RAPA (RAPA-) for 48 h. **b** Annexin V/PI flow cytometry data showing different apoptotic rates of WPMY-1 cells treated with 50 μM CQ (CQ+) or without CQ (CQ−) for 48 h. **c** Time kinetic curves showing that percentages of Annexin V+/PI− apoptotic WPMY-1 cells changed after 50 nM RAPA or 50 μM CQ treatment for 24, 48, and 72 h, respectively. **d**, **e** Annexin V/PI flow cytometry results showing different apoptotic rates of WPMY-AR cells cultured with 0 nM (DHT 0), 1 nM (DHT 1) and 10 nM (DHT 10) DHT, respectively, with 50 nM RAPA (RAPA+) or without RAPA (RAPA−) for 48 h. **f** Time kinetic curves showing that percentages of Annexin V+/PI− apoptotic WPMY-AR cells changed after treatment with DHT plus 50 nM RAPA for 24, 48, and 72 h, respectively. **g** Western blot data showing expression change of PARP, cleaved PARP (C-PARP), Caspase-3, and cleaved Caspase-3 (C-Caspase-3) levels as well as LC3 conversions in WPMY-1 cells after 50 μM CQ or 50 nM RAPA treatment for 24 and 48 h. **h** Western blot results showing expression change of PARP, C-PARP, Caspase-3, and C-Caspase-3 levels as well as LC3 conversions in WPMY-AR cells after treatment with different concentrations of DHT and RAPA (50 nM) treatment for 48 h. *P < 0.05, **P < 0.01

Since apoptosis reduction in prostate stromal fibroblasts is essential for BPH pathogenesis, we examined the apoptotic process with or without autophagy induction, in order to assess the association of autophagy with apoptosis in prostate stromal fibroblasts. To this end, we performed flow cytometry to determine Annexin V+/PI− apoptotic cells as well as Western blot to assess the apoptotic cascade and LC3 conversion ratio. After treatment with 50 nM RAPA for autophagy induction for 24, 48, and 72 h, the numbers of apoptotic cells were decreased notably compared with control values (Fig. 3a, c). More importantly, the expression levels of cleaved PARP and cleaved Caspase-3 in the apoptotic cascade were time-dependently decreased, while the LC3 conversion ratio was increased following RAPA treatment (Fig. 3g). In contrast, significantly more apoptotic cells were detected after treatment with 50 μM CQ for 24, 48, and 72 h, respectively (Fig. 3b, c, g). These results suggested that autophagy may promote viability of the prostatic fibroblasts by attenuating apoptotic process. To examine the role of AR signaling in the interrelationship between apoptosis and autophagy in prostatic fibroblasts, the apoptotic rate of WPMY-AR cells was assessed after treatment with 0, 1, and 10 nM DHT, respectively, plus 50 nM RAPA simultaneously. As shown in Fig. 3d,f, the apoptotic rate of WPMY-AR cells was increased in a dose and time-dependent manner after DHT treatment. Meanwhile, the expression levels of cleaved PARP and cleaved Caspase-3 remained very low after AR signal suppression (Fig. 3h). However, 50 nM RAPA, which induces LC3 conversion, attenuated DHT-induced apoptosis (Fig. 3e, f), and reduced cleaved PARP and cleaved Caspase-3 levels (Fig. 3h). These findings indicated that increased autophagy after androgen ablation could interfere with apoptosis in prostate stromal fibroblasts, which could contribute to the clinical progression of BPH.

### ATG9A is upregulated after AR signaling impairment

**Fig. 4:**
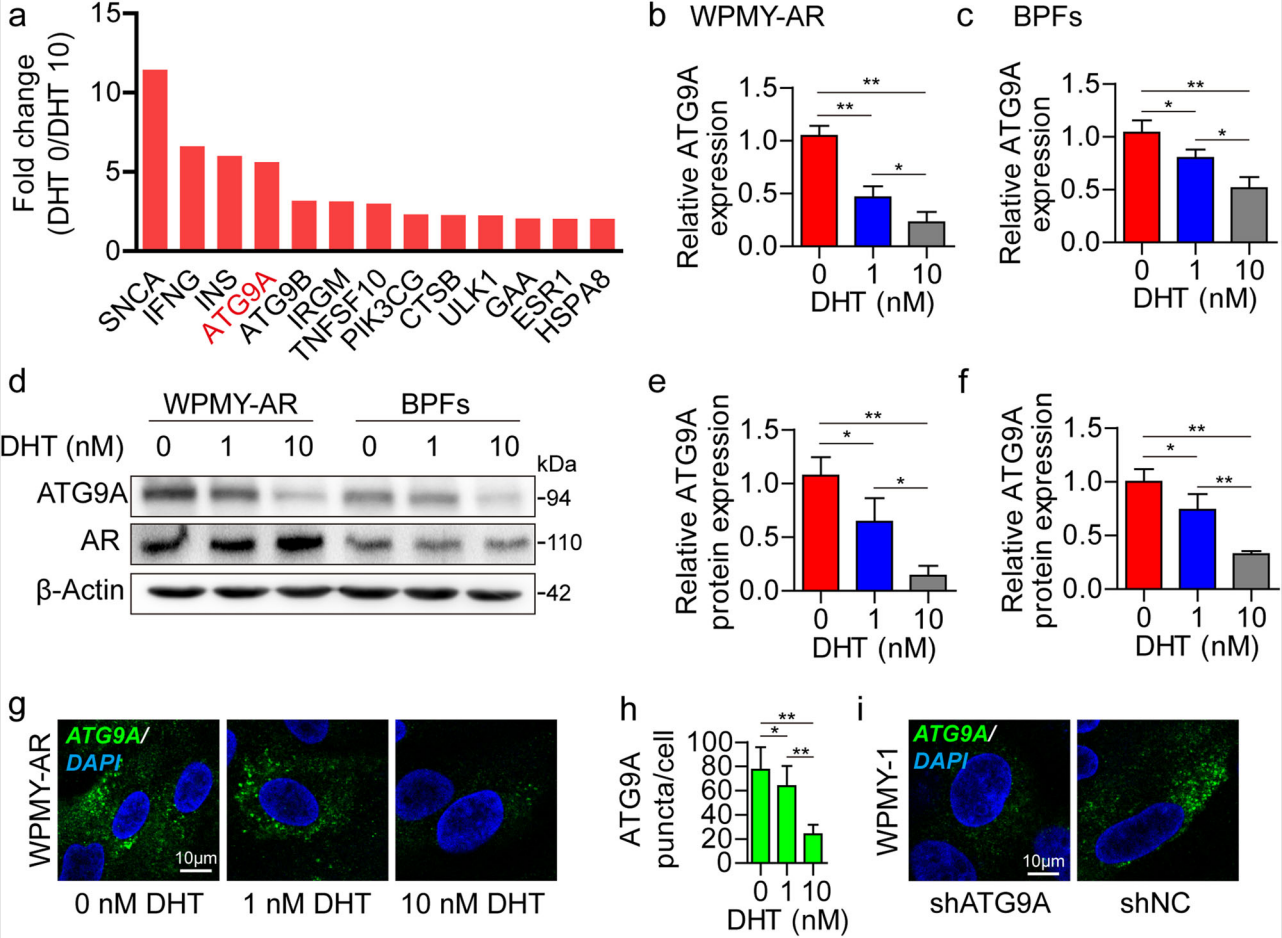
ATG9A expression change in prostate stromal fibroblasts after DHT treatment. **a** PCR array data showing fold change of differentially expressed ATG genes in WPMY-AR cells cultured with 0 nM DHT vs. 10 nM DHT (DHT 0/DHT 10). **b**, **c** qRT-PCR data showing ATG9A mRNA expression levels in WPMY-AR cells or BPFs after treatment with 0, 1, and 10 nM DHT for 48 h, respectively. **d** Western blot data showing ATG9A and AR protein expression levels in WPMY-AR cells (**e**) or BPFs (**f**) after treatment with 0, 1, and 10 nM DHT treatment for 48 h, respectively. **g**, **h** Immunofluorescent images showing ATG9A puncta accumulation in WPMY-AR cells treated with 0, 1, and 10 nM DHT, respectively. **i** Immunofluorescent images showing ATG9A puncta accumulation in WPMY-1 cells after knockdown of ATG9A (shATG9A). Scale bar, 10 μm. *P < 0.05, **P < 0.01

Previous studies reported that AR signaling participates in different steps of autophagy development based on normal or malignant prostate epithelial cells^18,19^. Few studies, however, focused on the interplay between AR signaling and ATG proteins in prostate stromal cells. Here we screened differentially expressed ATG genes after androgen ablation in WPMY-AR cells by PCR array (Supplementary Figure S1, and Supplementary Table 2). Via PCR array profiling, we found that ATG9A was overexpressed more than 5-fold higher at 0 nM DHT relative to 10 nM DHT (Fig. 4a). The PCR array results were verified by quantitative real-time polymerase chain reaction (qRT-PCR) in WPMY-AR and BPFs (Fig. 4b, c). Western blot (Fig. 4d–f) and immunofluorescence (IF) (Fig. 4g, h) further confirmed that the protein levels of ATG9A were reduced after DHT treatment in a dose-dependent manner. In human BPH tissues, ATG9A was upregulated in stromal fibroblasts, especially in tissues from patients administered 5-ARI (Supplementary Figure S2). These data revealed that ATG9A expression was suppressed by AR signaling activation and upregulated after androgen ablation. To further study the function of ATG9A in prostate stromal fibroblasts, we established ATG9A knockdown cells by shRNA via the lentivirus system (Fig. 4i, and Supplementary Figure S3a).

### ATG9A knockdown inhibits autophagic flux in prostate stromal fibroblasts

**Fig. 5:**
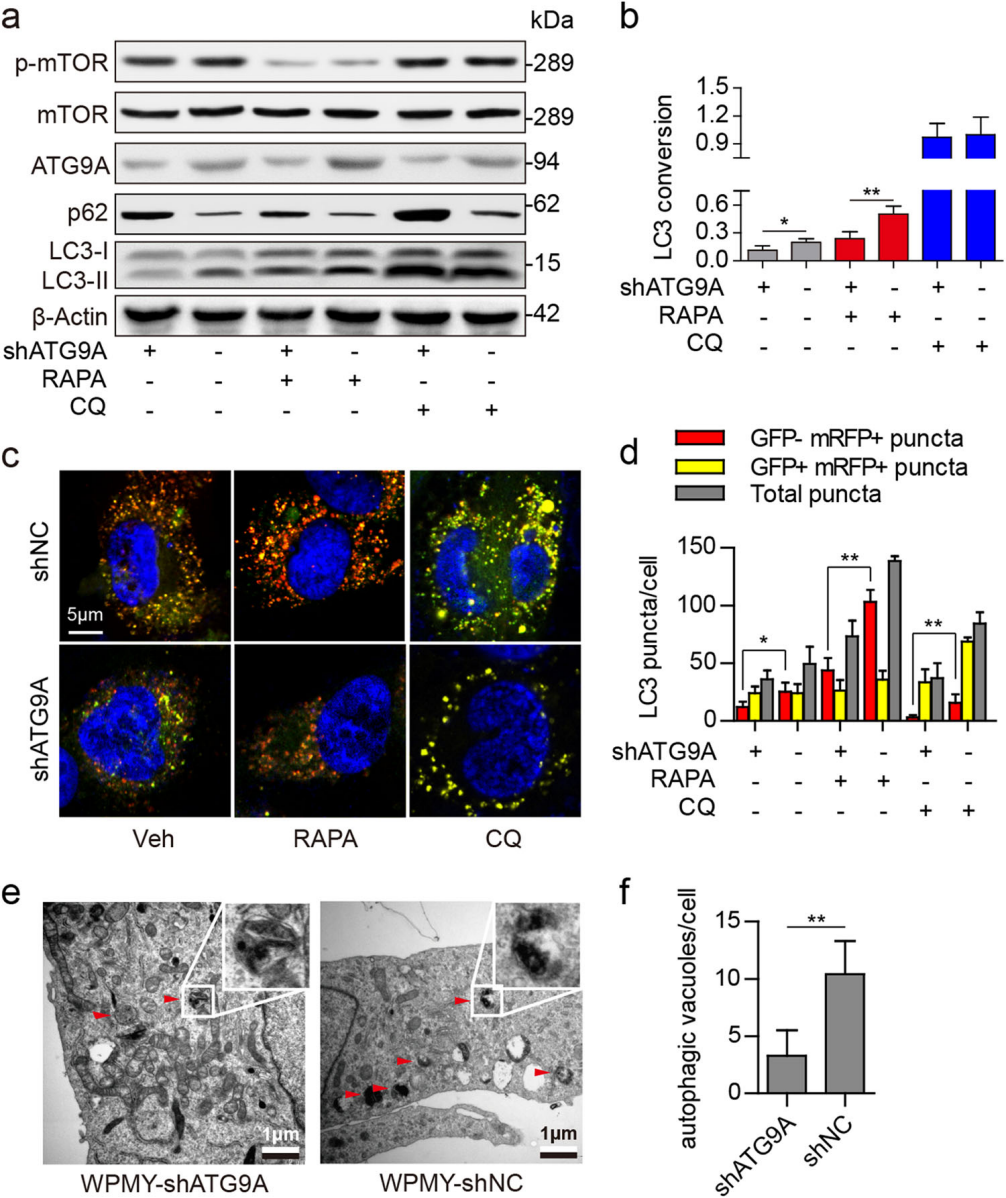
Autophagy in prostate stromal fibroblasts after ATG9A knockdown. **a**, **b** Western blot data showing phospho-mTOR (p-mTOR), mTOR, ATG9A, and p62 protein expression levels and the LC3 conversion ratio (LC3-II/β-Actin) in WPMY-1 cells after ATG9A knockdown. The cells were treated with 50 nM RAPA or 50 μM CQ for 3 h before protein extraction. **c**, **d** ATG9A knockdown WPMY-1 cells were infected with mRFP–GFP–LC3 adenovirus for 48 h, and treated with 50 nM RAPA or 50 μM CQ for 3 h before 4% paraformaldehyde fixation and DAPI counterstaining. Scale bar, 5 μm. Bar graphs showing LC3 puncta formation in different groups. **e**, **f** Transmission electron micrographs for autophagic vacuoles detection in ATG9A knockdown WPMY-1 (WPMY-shATG9A) or control (WPMY-shNC) cells. The cells were treated with 50 nM RAPA for 3 h before 2% glutaraldehyde fixation. Enlarged images show double-membrane autophagic vacuoles. Scale bar, 1 μm. *P < 0.05, **P < 0.01

It is known that mammalian ATG9A is essential for phagophore assembly and autophagosome formation through induction of membrane trafficking between the Golgi apparatus and the endosomes^20,21^. In this study, ATG9A knockdown by shRNA triggered p62 level increase but repressed LC3 conversion in WPMY-1 and BPFs, with no effects on mTOR protein levels or phosphorylation (Fig. 5a, b, and Supplementary Figure S3a, b). Moreover, the mRFP–GFP–LC3 system further reported that the numbers of all LC3 puncta (yellow + red puncta) or autolysosomes (red puncta) were decreased after ATG9A knockdown in WPMY-1 cells and in BPFs (Fig. 5c, d, and Supplementary Figure S3c, d). In addition, TEM revealed fewer autophagic vacuoles in ATG9A knockdown WPMY-1 cells (WPMY-shATG9A) compared with control cells (WPMY-shNC) (Fig. 5e, f). These findings suggested that ATG9A was essential for regulating autophagic flux in prostate stromal fibroblasts. Next, we examined autophagic flux after ATG9A knockdown in WPMY-AR cells under different concentrations of DHT. The results demonstrated that androgen ablation could not induce autophagy as much as before ATG9A knockdown (Supplementary Figure S4). These data revealed that androgen ablation induced ATG9A upregulation was an important reason for autophagy escalation in prostate stromal fibroblasts.

### ATG9A knockdown in prostate stromal fibroblasts inhibits the growth of recombinant grafts in *nude* mice

**Fig. 6:**
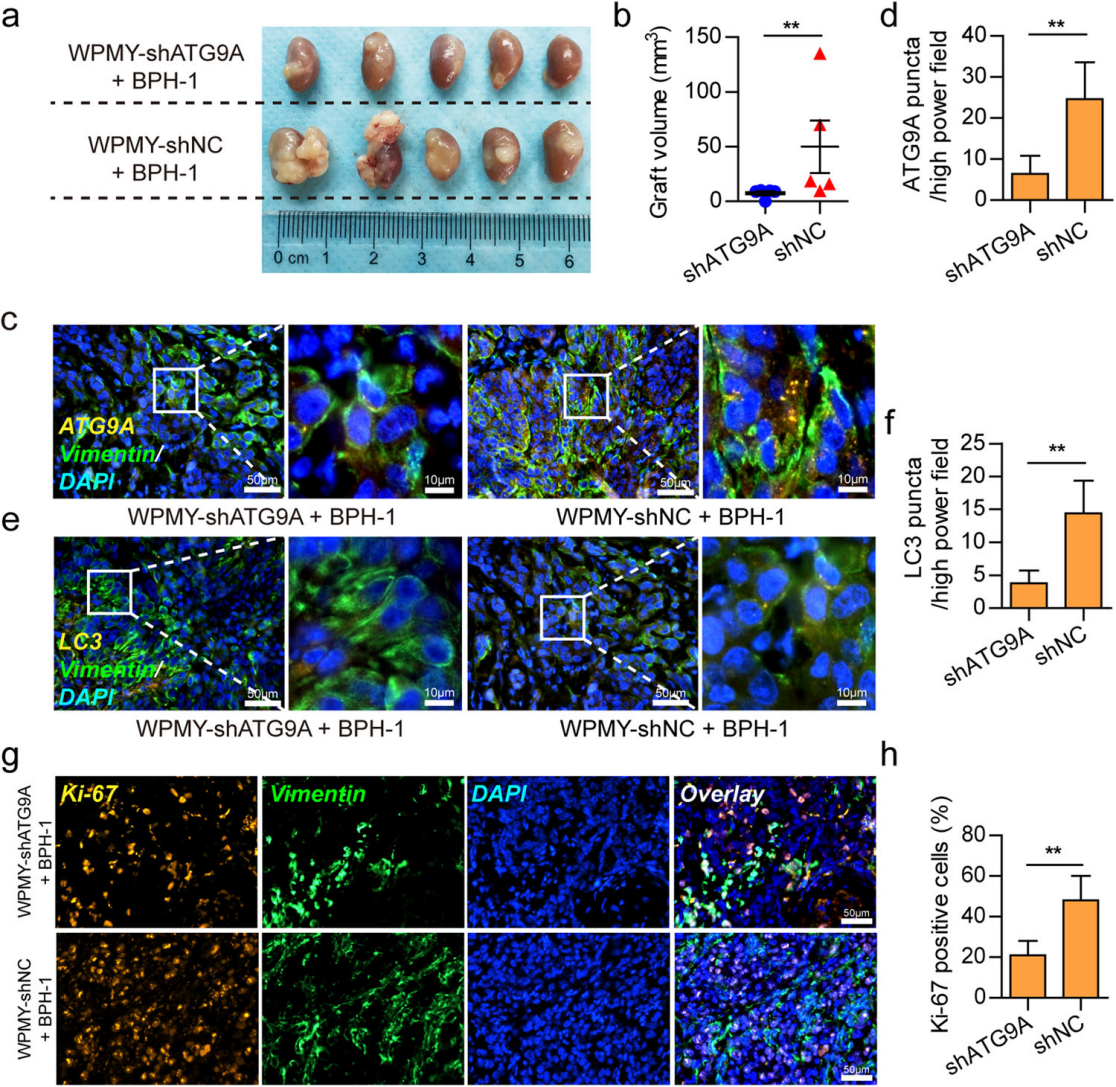
Prostatic epithelial cell and stromal fibroblast recombination under the renal capsule in *nude* mice. **a**, **b** Recombinant grafts in each group. The shATG9A group (WPMY-shATG9A+BPH-1) showed reduced volumes of recombinant grafts compared with the control group (WPMY-shNC+BPH-1). **c**, **d** Immunofluorescent images showing ATG9A and Vimentin signals in the recombinant grafts of different groups. Bar graph showing the numbers of ATG9A puncta per high power field in different groups. **e**, **f** Immunofluorescent images showing LC3 and Vimentin signals in recombinant grafts of different groups. Bar graph showing the numbers of LC3 puncta per high power field in different groups. **g**, **h** Immunofluorescent images showing Ki-67 and Vimentin signals in recombinant grafts of different groups. Bar graph showing proportions of Ki-67 positive cells in different groups. Scale bar, 50 μm in low power images or 10 μm in high-power images. **P < 0.01

To examine the effects of ATG9A knockdown in prostate stromal fibroblasts on the BPH tissue microenvironment, we established prostatic fibroblast and epithelial cell recombination under the renal capsule in *nude* mice. One week prior to graft implantation, all animals were administered finasteride for androgen ablation, mimicking BPH patients with 5-ARI treatment. After 12 weeks, the animals’ kidneys were excised. As shown in Fig. 6a, b, the shATG9A group (WPMY-shATG9A+BPH-1) showed notably reduced graft volumes compared with the control group (WPMY-shNC+BPH-1). Moreover, Vimentin positive stromal fibroblasts in the shATG9A group showed fewer ATG9A puncta (Fig. 6c, d) and assembled less LC3 puncta, compared with the shNC group (Fig. 6e, f). LC3 was dispersed in the cytoplasm without puncta formation in the shATG9A group, confirming that autophagy was inhibited by ATG9A knockdown. In addition, the shATG9A group showed fewer Ki-67-positive epithelial cells and a reduced proportion of Vimentin positive stromal compartment compared with the shNC group (Fig. 6g, h). These findings suggested that ATG9A knockdown inhibited the development of prostate epithelial cells and stromal fibroblasts.

## Discussion

This study revealed that the increased autophagy level in prostate stromal fibroblasts is an important feature of androgen ablation in BPH tissues. Upregulation of ATG9A by impaired AR signaling after long-term 5-ARI administration contributed to autophagy level elevation and promoted BPH progression. Knockdown of ATG9A in prostate stromal fibroblasts reduced not only the prostatic fibroblasts’ autophagy levels but also the growth of recombinant grafts in vivo.

Morphometric analyses revealed that the enlarged prostate is featured by hyperplastic epithelial and stromal compartments^22,23^. The balance of stromal-epithelial crosstalk in the prostate has essential physical and functional meaning for prostate development. However, in prostatic diseases, such as BPH, prostatitis, and prostate cancer, homeostasis between stromal and epithelial compartments in the tissue microenvironment is broken^22,24^. The stromal compartment that consists mostly of fibroblasts can occupy more than 70% of BPH tissues, suggesting that hyperproliferation of stromal fibroblasts is an essential mechanism in the pathological process of BPH^25^. The prostate is an androgen responsive organ, indicating that AR signaling is essential for homeostasis and pathology in the prostate^3,26^. Testosterone is the main circulating androgen in men but has significantly lower potential to stimulate prostate growth compared with DHT, which is converted from testosterone by type II 5 α-reductase in prostate cells. 5-ARIs, such as finasteride and dutasteride, inhibit DHT synthesis by targeting 5α-reductase, and can be used to reduce prostate size, relieve BPH induced LUTS, and BPH-related hematuria^7,9,27^. After long-term treatment, however, BPH may still progress and some patients have to undergo surgical interventions or catheterization in the latent state^28^. Unfortunately, few studies have explored the underlying mechanisms of BPH progression after long-term 5-ARI treatment.

Inhibition of AR signaling induces autophagy in prostate cancer^19^. It is worth assessing whether androgen ablation induces autophagy in BPH. Previously, we found a notable correlation between long-term 5-ARI treatment and high expression levels of LC3 and Beclin-1, which are considered autophagy indicators in both the stromal and epithelial compartments of BPH tissues^17^. More importantly, the present study showed that autophagy induced by rapamycin reduced apoptosis in prostate stromal fibroblasts. These findings indicate that autophagy plays a vital role in the proliferation of prostate stromal fibroblasts and BPH progression after long-term 5-ARI treatment.

Currently, function and regulation of autophagy are largely understood^29^. It was suggested that autophagy affects cell death in response to various aspects, such as nutrient starvation and hypoxia. Accumulating evidences indicate that autophagy is regulated by various ATG proteins. In the present study, ATG9A was deregulated after androgen ablation in prostate stromal fibroblasts. ATG9A is an integral membrane protein that involves in autophagosome formation^30^. It is found between the phagophore assembly site (PAS)/pre-autophagosomal structure and peripheral sites like the Golgi apparatus^31–33^, and essential for autophagy initiation. The mechanisms of ATG9A trafficking remain unclear, although previous reports have revealed that ATG9A has an important effect on autophagosome formation^20,21,34^. The mechanisms of ATG9A in autophagy also play vital roles in chemotherapy resistance^35^ and disease recurrence^36^ in certain malignancies. In addition, microRNAs targeting the ATG9A mRNA inhibit autophagy and constitute potential therapeutic tools in pancreatic cancer, acute kidney injury, and cardiac hypertrophy^37–39^. The current study confirmed that ATG9A knockdown by shRNA inhibited autophagy in prostate stromal fibroblasts, indicating that ATG9A also plays vital roles in regulating autophagy in prostatic hyperplasia.

How does androgen-AR signaling regulate ATG9A expression level remains unclear. In our study, we found that the AR activation by DHT in prostate stromal fibroblasts repressed ATG9A expression. The possible mechanisms underlying this process may due to the genomic regulation or non-genomic actions of AR. Since AR is a ligand-regulated transcription factor, its major function is bind to androgen response elements (AREs) on promoter area of a gene. AREs can be classified into positive AREs (pAREs) mediating the transcriptional activation of AR target genes, and negative AREs (nAREs) mediating an AR-dependent repression^40^. Whether nAREs that exist around ATG9A transcriptional area in prostatic fibroblasts need further study. Another mechanism of AR-mediated gene repression is the inhibition of transactivating factors by AR which decreases the DNA binding ability or gene-activating function of the transcription factors^40^. Thus, AR may also mediate ATG9A repression by interacting with and inhibiting the functions of transcription factors which promotes ATG9A transcription. Furthermore, other reasons such as the non-genomic actions of AR which lead to gene repression cannot be excluded. For example, AR interplays with Hic-5 in cytoplasm of prostate myofibroblasts which facilitates Hic-5 nuclear translocation, thereby induces the gene regulatory functions of Hic-5^41^; AR also leads to p27 Ser10 phosphorylation and cell cycle arrest through androgen-triggered AR/filamin A complex in NIH3T3 fibroblast model^42^. Therefore, AR may also regulate ATG9A gene expression and autophagy level through non-genomic mechanisms. Anyhow, AR ablation-induced ATG9A deregulation is one of the important reasons for the increase of autophagy level in the prostate stromal fibroblasts after long-term 5-ARI administration. To understand the mechanisms underlying this process need further experiments.

In conclusion, findings in this study indicate that ATG9A upregulation after long-term 5-ARI treatment induces autophagy in prostate stromal fibroblasts, constituting an essential reason for BPH progression. Hence, in addition to receiving 5-ARI therapy, other approaches, such as inhibition of autophagy via targeting ATG9A, may become an effective method to reduce the risk of BPH progression.

## Materials and methods

### Antibodies and reagents

Anti-LC3 (#12741), mTOR (#2983), phospho-mTOR (Ser2448) (#5536), PARP (#9542), and Caspase-3 (#9665) antibodies were from Cell Signaling Technology (Danvers, MA, USA). Anti-Beclin-1 (ab62472), ATG9A (ab108338), Ki-67 (ab15580), and Vimentin (ab8978) antibodies were from Abcam (Cambridge, UK). Anti-β-Actin (D110001) antibody was from Sangon Biotech (Shanghai, China). Alexa Fluor 488-conjugated goat anti-mouse IgG and Alexa Fluor 555-conjugated goat anti-rabbit IgG were manufactured by Jackson ImmunoResearch (West Grove, PA, USA). DHT was purchased from Sigma-Aldrich (St Louis, MO, USA) and dissolved in ethanol. RAPA, CQ, and finasteride were from Selleck (Shanghai, China). RAPA and finasteride were dissolved in dimethyl sulfoxide (DMSO) (Sigma-Aldrich); CQ was dissolved in ultrapure water.

### Prostate samples

Prostate samples were obtained from transurethral resection of the prostate or radical prostatectomy at Shanghai General Hospital (Shanghai, China), and confirmed by pathology. Detailed information of the patients is shown in Supplementary Table 3. For experiments involving human subjects, approval was obtained from the institutional review board of Shanghai General Hospital ethics committee. Written informed consent was obtained from all patients.

### Cell culture

BPH-derived prostate fibroblasts (BPFs) and normal prostate fibroblasts (NPFs) were obtained from prostate samples and cultured as described previously^43^. WPMY-1 (immortalized prostate stromal fibroblasts, ATCC, Manassas, VA, USA) and BPH-1 (immortalized prostate epithelial cell line, preserved in Laboratory of Urology Center, Shanghai General Hospital) were cultured in Dulbecco’s modified Eagle’s medium (DMEM) (HyClone, South Logan, UT, USA) containing 5% fetal bovine serum (FBS) (Gibco, Grand Island, NY, USA) and 1% penicillin–streptomycin solution (Gibco). All cells were cultured in a humidified atmosphere containing 5% CO_2_ at 37 °C. Before DHT treatment, cells were DHT starved by culture in phenol red-free DMEM (Gibco) containing charcoal stripped FBS (Gibco) for 3 days.

### AR overexpression in WPMY-1 cells

The coding sequence (CDS) of the AR mRNA (NM_000044) was synthesized and cloned into pLV-CMV-3× flag-puro lentivirus overexpression vectors. After packaging, pLV-AR-3× flag-puro was used to infect WPMY-1 cells at a multiplicity of infection (MOI) of 30, for the construction of AR overexpressing WPMY-AR cells.

### mRFP–GFP–LC3 adenovirus infection

The mRFP–GFP–LC3 adenovirus was purchased from Genomeditech (Shanghai, China) and used to track autophagic flux. After adenovirus infection, the cells are able to express mRFP–GFP-labeled LC3 protein. The GFP signal is sensitive to the acidic conditions of the lysosomal lumen, whereas mRFP is stable. Therefore, yellow puncta (GFP+ and mRFP+) indicate the presence of autophagosomes. In contrast, red puncta (GFP− and mRFP+) correspond to autolysosomes. Autophagic flux was monitored by quantifying yellow and red LC3 puncta accumulation. After adenovirus infection for 48 h, the cells were treated with 50 nM RAPA or 50 μM CQ for 3 h and imaged under a Leica TCS SP8 confocal microscope (Wetzlar, Germany). At least 10 cells in each group were analyzed.

### Knockdown of ATG9A

Short-hairpin RNA (shRNA) targeting ATG9A mRNA was designed as described previously^32^ and cloned into pGLV2 (GenePharma, Shanghai, China) shRNA lentivirus vectors. After packaging, the lentivirus was used to infect prostate stromal fibroblasts for ATG9A knockdown.

### Apoptosis assay

Apoptosis was assessed with FITC Annexin V apoptosis detection kit (BD Biosciences, San Diego, CA, USA) according to the manufacturer’s instructions, on BD Accuri C6 flow cytometer (BD Biosciences).

### qRT-PCR and PCR array

Total cellular RNA was extracted with TRIzol reagent (Life Technologies, Foster City, CA, USA). Reverse transcription was carried out with PrimeScript RT Master Mix (Takara, Otsu, Shiga, Japan) according to the manufacturer’s instructions. Then, qRT-PCR was performed with SYBR Premix Ex Taq (Takara). GAPDH was used as an internal control. The following primers were used: ATG9A, forward 5′-TCCTCTTTGTGGTTGCCTTC-3′ and reverse 5′-AGTGACCTTGACGGGTTCAG-3′; GAPDH, forward 5′-GAAGGTCGGAGTCAACGGATT-3′ and reverse 5′-CGCTCCTGGAAGATGGTGAT-3′. RT² Profiler PCR Array (Qiagen, Hilden, Germany) was used for assessing the expression levels of ATG genes in prostate stromal fibroblasts after treatment with different DHT concentrations. Relative mRNA expression was calculated by 2^−ΔΔCt^ method.

### Western blot

For protein expression assessment, the tissues were lysed with RIPA buffer (Beyotime, Suzhou, China). The proteins were separated by SDS-polyacrylamide gel electrophoresis and transferred onto polyvinylidene fluoride membrane (Millipore, Billerica, MA, USA). The signals were detected by ChemiLucent ECL Detection system (Millipore) and quantitated by the ImageJ software (National Institutes of Health). The LC3 conversion ratio (LC3-II/β-Actin) was determined to monitor autophagy levels.

### Transmission electron microscopy

To assess autolysosome formation, the cells were treated with 50 nM RAPA for 3 h, and collected for TEM analysis. Ultrathin sections were examined on a CM-120 electron microscope (Philip, Eindhoven, Netherlands).

### In vivo cells recombination

In vivo recombinant grafts were made by mixing WPMY-1 and BPH-1 cells in a ratio of 1:1; 6-to-8-week-old male *nude* mice (Animal Center of the Chinese Academy of Sciences, Shanghai, China) were used. All mice received 20 mg/kg finasteride once daily, subcutaneously 1 week before the graft implantation. A total of 2 × 10^5^ mixed cells were resuspended in 50 μl phosphate-buffered saline (PBS) (HyClone) 1:1 diluted Matrigel (Corning, New York, NY, USA) and injected under the renal capsule. Twelve weeks later, the mice were sacrificed and sub-renal capsule grafts were collected for IF.

### IHC and IF

IHC and IF were performed as described previously^17,24^. IHC images were acquired under a Leica DM5500 B microscope. IF images of cells and tissues were acquired under Leica TCS SP8 confocal microscope and Leica DMi8 fluorescence microscope, respectively.

### Statistics

Statistical analyses were carried out with the SPSS software (version 19, SPSS Inc., Chicago, IL). All experiments were repeated three times. Data are mean ± standard deviation (SD). Differences between groups were analyzed by two-sided Student’s *t*-test or one-way ANOVA. Statistical significance was set at *P < *0.05.

## Electronic supplementary material

supplementary figure1

supplementary figure2

supplementary figure3

supplementary figure4

Supplementary Table 1 and 3

Supplementary Table 2

Supplementary Information
