# Supplementary figures and images for: Deregulation of ATG9A by impaired AR signaling induces autophagy in prostate stromal fibroblasts and promotes BPH progression
Source: Cell Death Dis. 2018 Mar 22;9(4):431. doi: 10.1038/s41419-018-0415-2 (PMC5864884; doi:10.1038/s41419-018-0415-2)

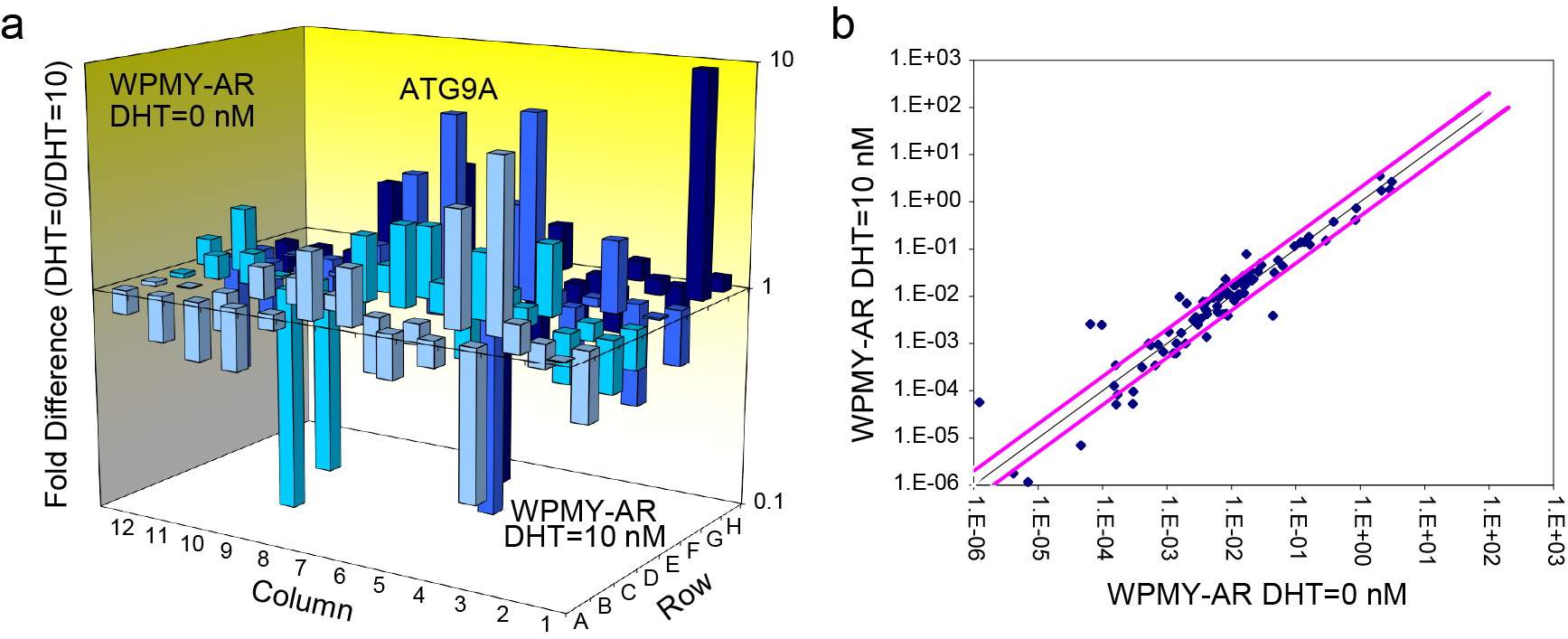

Supplement: Supplementary file 1 — supplementary figure1 [file 41419_2018_415_MOESM1_ESM.tif]

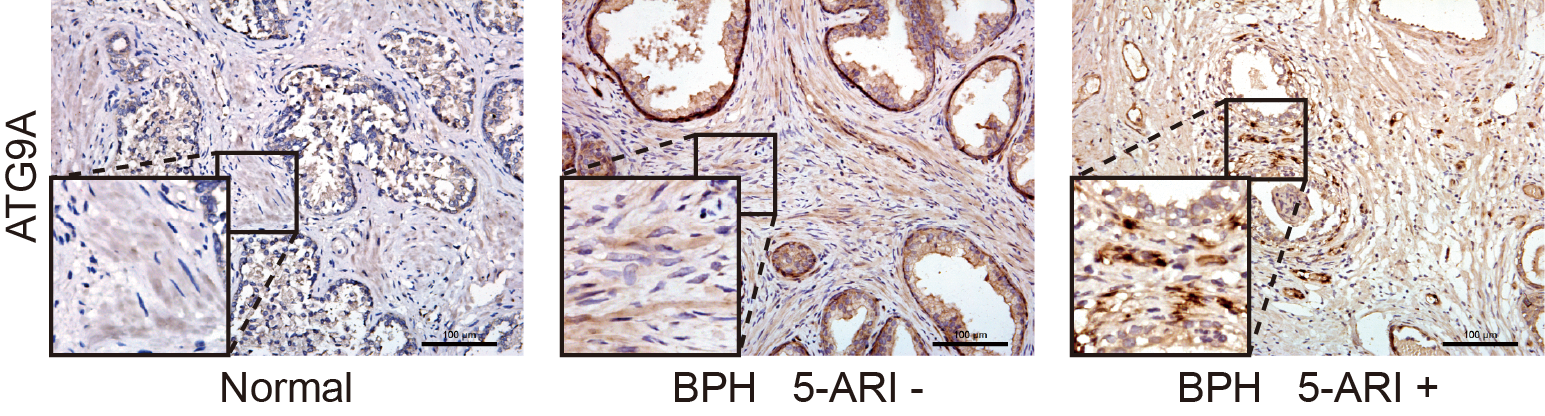

Supplement: Supplementary file 2 — supplementary figure2 [file 41419_2018_415_MOESM2_ESM.tif]

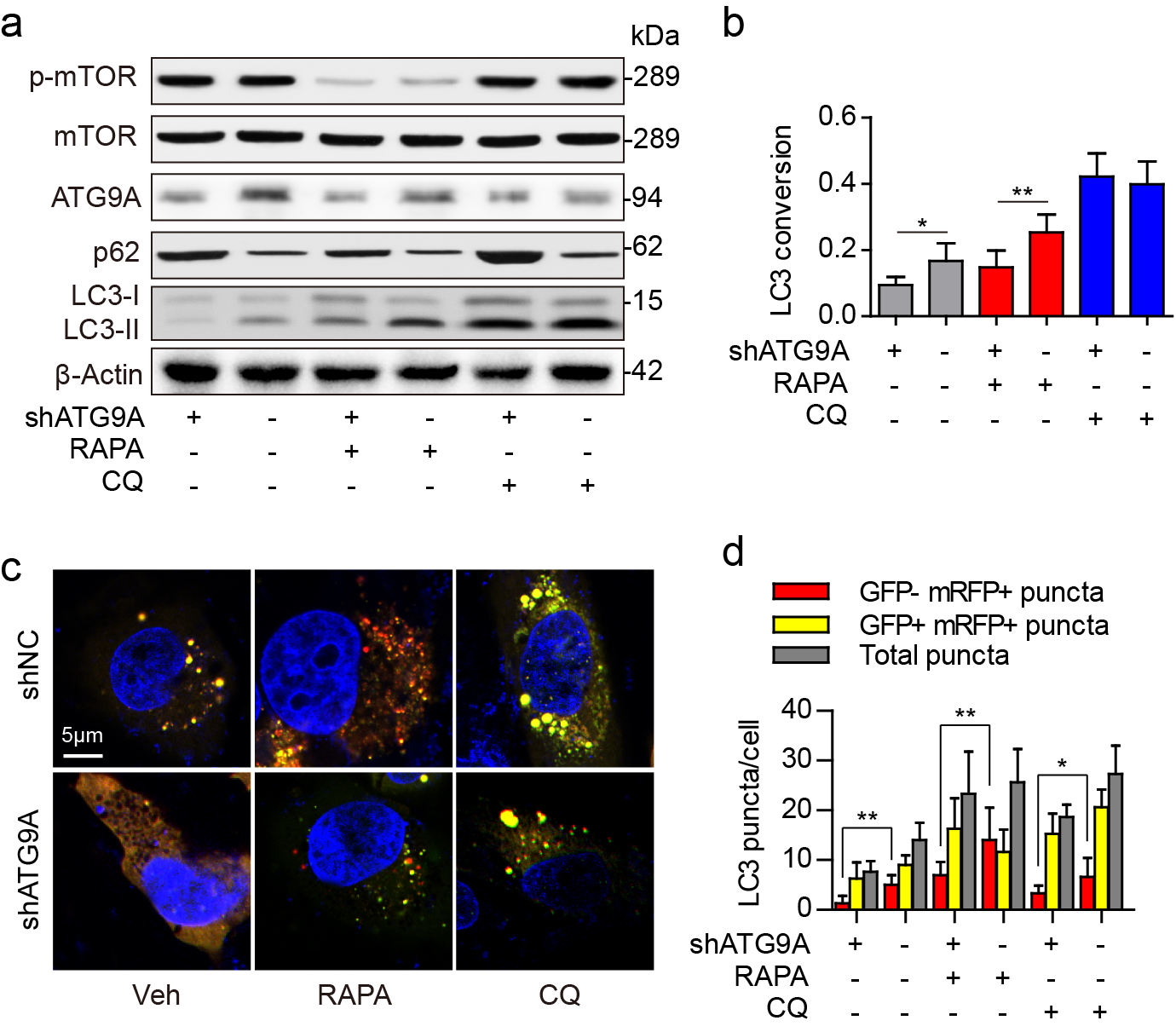

Supplement: Supplementary file 3 — supplementary figure3 [file 41419_2018_415_MOESM3_ESM.tif]

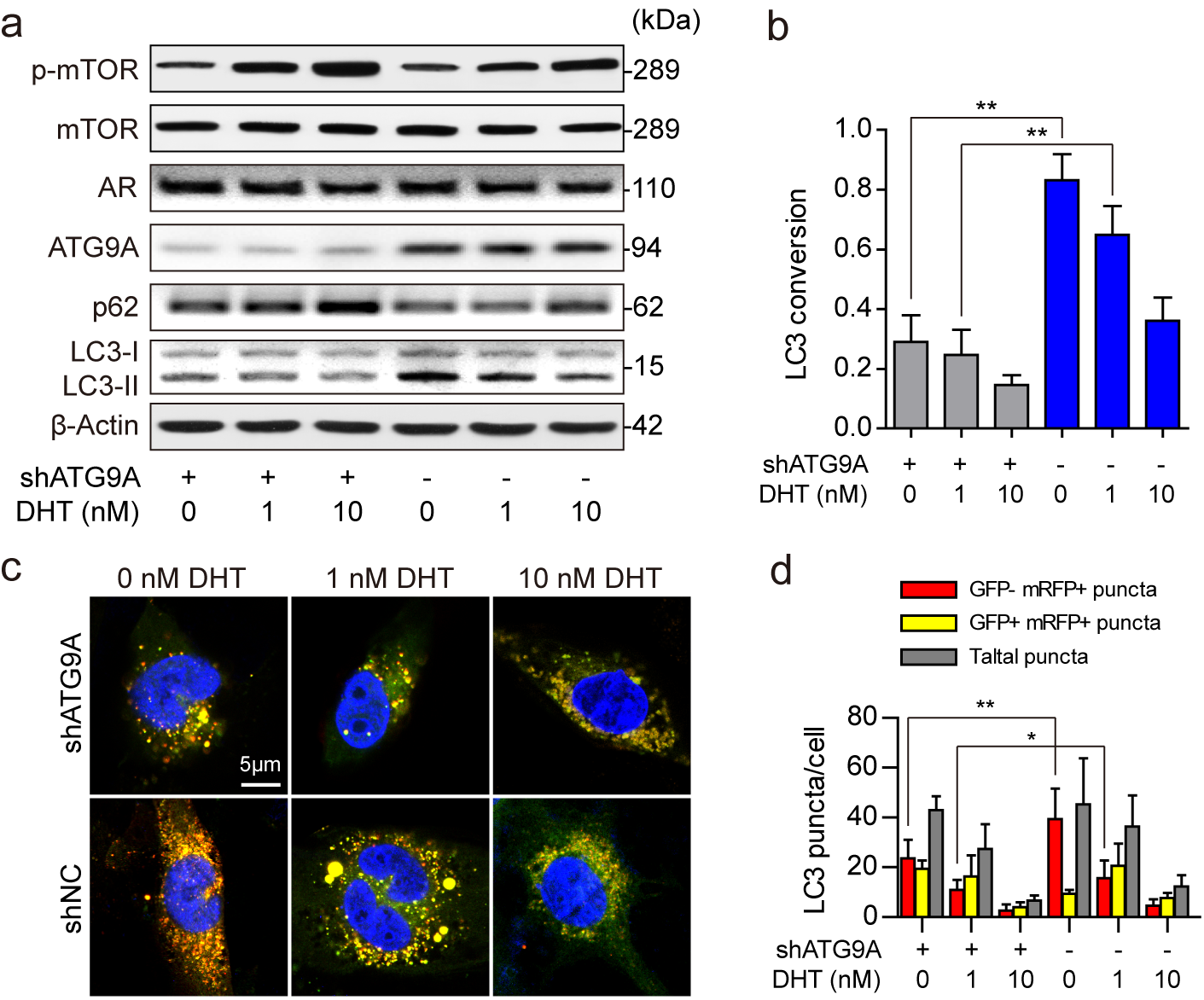

Supplement: Supplementary file 4 — supplementary figure4 [file 41419_2018_415_MOESM4_ESM.tif]
