# Supplementary material for: Deregulation of ATG9A by impaired AR signaling induces autophagy in prostate stromal fibroblasts and promotes BPH progression
Source: Cell Death Dis. 2018 Mar 22;9(4):431. doi: 10.1038/s41419-018-0415-2 (PMC5864884; doi:10.1038/s41419-018-0415-2)
Supplement: Supplementary file 5 — Supplementary Table 1 and 3 [file 41419_2018_415_MOESM5_ESM.docx]

**Supplementary Tables:**

**Supplementary Table 1** Contingency table showing the numbers of patients in various groups and IHC score stratification. The IHC score was defined according to LC3 or Beclin-1 staining intensity in the stromal compartment of the prostate tissue.

| IHC score | Normal (n=30) | BPH 5-ARI – (n=30) | | BPH 5-ARI + (n=30) | *P* value |
| --- | --- | --- | --- | --- | --- |
| Stromal LC3 expression |  | |  |  | < 0.01 ^a^ |
| 1 | 16 (53.3 %) | | 1 (3.3 %) | 0 (0.0 %) |  |
| 2 | 13 (43.3 %) | | 4 (13.3 %) | 3 (10.0 %) |  |
| 3 | 1 (3.3 %) | | 15 (50.0 %) | 10 (33.0 %) |  |
| 4 | 0 (0.0 %) | | 10 (33.0 %) | 17 (56.7 %) |  |
| Stromal Beclin-1 expression |  | |  |  | 0.039 ^a^ |
| 1 | 13 (43.3 %) | | 11 (36.7 %) | 7 (23.3 %) |  |
| 2 | 12 (40.0 %) | | 9 (30.0 %) | 6 (20.0 %) |  |
| 3 | 5 (16.7 %) | | 7 (23.3 %) | 10 (33.0 %) |  |
| 4 | 0 (0.0 %) | | 3 (10.0 %) | 7 (23.3 %) |  |
| IHC, immunohistochemistry; BPH, benign prostatic hyperplasia; 5-ARI -, without 5α-reductase inhibitor treatment; 5-ARI +, with 5α-reductase inhibitor treatment.  ^a^ Fisher’s exact test. | | | | | |

**Supplementary Table 3** Patients’ clinical parameters.

| Clinical  Parameters | Normal (n=30) | | BPH 5-ARI - (n=30) | | BPH 5-ARI + (n=30) | | *P* value |
| --- | --- | --- | --- | --- | --- | --- | --- |
|  | Mean ± SD | Range | Mean ± SD | Range | Mean ± SD | Range |  |
| Age (year) | 70.72 | 64-79 | 69.06 ± 8.46 | 53-82 | 71.06 ± 7.39 | 55-87 | 0.735 |
| PV (ml) | 23.6 ± 5.71 | 16.63-35.72 | 59.39 ± 35.91 | 27.77-99.28 | 66.58 ± 41.71 | 25.96-153.69 | <0.01 ^a^/0.23 ^b^ |
| PSA (ng/ml) | NA | NA | 9.69 ± 8.22 | 1.79-23.7 | 5.83 ± 6.48 | 0.30-19.28 | 0.014 |
| IPSS | NA | NA | 20.15 ± 5.76 | 9-30 | 19.68 ± 5.23 | 10-30 | 0.657 |
| QoL | NA | NA | 4.7 ± 0.85 | 3-6 | 4.5 ± 1.07 | 3-6 | 0.781 |
| Qmax (ml/s) | NA | NA | 8.33 ± 3.72 | 4.01-18.70 | 10.38 ± 4.85 | 7.86-21.69 | 0.084 |
| PVR (ml) | NA | NA | 86.70 ± 83.82 | 29.76-631.70 | 91.86 ± 89.77 | 33.04-762.15 | 0.136 |
| BPH, benign prostatic hyperplasia; 5-ARI -, without 5α-reductase inhibitor treatment; 5-ARI +, with 5α-reductase inhibitor treatment; SD, standard deviation; PV, prostate volume; PSA, prostate specific antigen; IPSS, international prostate symptom score; QoL, quality of life; Qmax, maximum urinary flow rate; PVR, post-void residual; NA, not available.  ^a^ Difference among the 3 groups.  ^b^ Difference between BPH 5-ARI - group and BPH 5-ARI + group. | | | | | | | |
