# Supplementary material for: Deregulation of ATG9A by impaired AR signaling induces autophagy in prostate stromal fibroblasts and promotes BPH progression
Source: Cell Death Dis. 2018 Mar 22;9(4):431. doi: 10.1038/s41419-018-0415-2 (PMC5864884; doi:10.1038/s41419-018-0415-2)
Supplement: Supplementary file 7 — Supplementary Information [file 41419_2018_415_MOESM7_ESM.docx]

**Supplementary materials**

**Supplementary Figure S1 Results of autophagy PCR array.** **a** Column graph illustrating fold changes of ATG genes in WPMY-AR cells treated with 0 nM DHT vs. 10 nM DHT (0 nM DHT/10 nM DHT). **b** Scatter plot of PCR array data comparing expression differences of ATG genes between 0 nM DHT and 10 nM DHT treatment.

**Supplementary Figure S2** Immunohistochemistry data showing ATG9A expression in normal prostate and BPH tissues after 5-ARI treatment (5-ARI+) or without 5-ARI treatment (5-ARI-). Enlarged images show different ATG9A expression levels in prostate stromal fibroblasts of various groups. Scale bar, 100 μm.

**Supplementary Figure S3 Autophagy in prostate stromal fibroblasts after ATG9A knockdown.** **a** Western blot data showing phospho-mTOR (p-mTOR), mTOR, ATG9A, and p62 protein expression levels as well as the LC3 conversion ratio (LC3-II/β-Actin) **b** in BPFs after ATG9A knockdown. The cells were treated with 50 nM RAPA or 50 μM CQ for 3 h before protein extraction. **c**, **d** ATG9A knockdown BPFs were infected with mRFP-GFP-LC3 adenovirus for 48 h, and treated with 50 nM RAPA or 50 μM CQ for 3 h before 4% paraformaldehyde fixation and DAPI counterstaining. Scale bar, 5 μm. Bar graphs showing LC3 puncta formation in different groups. * *P* < 0.05, ** *P* < 0.01.

**Supplementary Figure S4 ATG9A knockdown reduces androgen ablation induced autophagy in WPMY-AR cells. a** Western blot data showing phospho-mTOR (p-mTOR), mTOR, AR, ATG9A, and p62 protein expression levels as well as the LC3 conversion ratio (LC3-II/β-Actin) **b** in ATG9A knockdown or control WPMY-AR cells treated with different concentrations (0 nM, 1 nM, and 10 nM) of DHT. **c, d** ATG9A knockdown or control WPMY-AR cells were infected with mRFP-GFP-LC3 adenovirus and cultured with 1 nM and 10 nM DHT for 48 h, respectively, fixed with 4% paraformaldehyde, and counterstained with DAPI. Scale bar, 5 μm. Bar graph showing LC3 puncta formation in different groups.

**Supplementary Table 1** Contingency table showing the numbers of patients in various groups and IHC score stratification. The IHC score was defined according to LC3 or Beclin-1 staining intensity in the stromal compartment of the prostate tissue.

**Supplementary Table 2** Results of autophagic PCR array profiling.

**Supplementary Table 3** Patients’ clinical parameters.
